# Two-Layer Nanocomposite TiC-Based Coatings Produced by a Combination of Pulsed Cathodic Arc Evaporation and Vacuum Electro-Spark Alloying

**DOI:** 10.3390/ma13030547

**Published:** 2020-01-23

**Authors:** Philipp Kiryukhantsev-Korneev, Alina Sytchenko, Alexander Sheveyko, Dmitry Moskovskikh, Stepan Vorotylo

**Affiliations:** Scientific-Educational Center of SHS, National University of Science and Technology “MISiS”, 119049 Moscow, Russia; alina-sytchenko@yandex.ru (A.S.); sheveyko@mail.ru (A.S.); mosskiller@yandex.ru (D.M.); stepan.vorotylo@gmail.com (S.V.)

**Keywords:** titanium carbide, diamond-like carbon (DLC), NiCr, europium oxide, steel, vacuum electro-spark alloying, pulsed cathodic arc evaporation, hybrid VESA–PCAE technology, Ar and C_2_H_4_ environments, wear- and corrosion resistance

## Abstract

A novel two-stage technology combining vacuum electro-spark alloying (VESA) and pulsed cathodic arc evaporation (PCAE) was approbated for the deposition of TiC-based coatings in inert (Ar) and reactive (C_2_H_4_) atmospheres. The deposition was carried out using a TiC-NiCr-Eu_2_O_3_ electrode and 5140 steel substrates. Structural, elemental, and phase compositions of the deposited coatings were investigated by scanning electron microscopy, energy-dispersive spectrometry, and X-ray diffraction. The mechanical properties of the coatings were measured by nanoindentation using a 4 mN load. The tribological properties of the coatings were measured using the pin-on-disc setup in air and in distilled water at a 5 N load. The experimental data suggest that VESA coatings are characterized by surface defects, a hardness of 12.2 GPa, and a friction coefficient of 0.4. To ensure good adhesion between the VESA coating and the upper layer containing diamond-like carbon (DLC), an intermediate layer was deposited by PCAE in the Ar atmosphere. The intermediate layer had a hardness of up to 31 GPa. The upper layer of the coating ensured a low and stable friction coefficient of 0.2 and high wear resistance due to the formation of an sp^2^–sp^3^ bound carbon phase. Multilayer TiC-based coating with the upper DLC layer, in addition to high tribological properties, was characterized by the lowest corrosion current density (12 μA/cm^2^).

## 1. Introduction

The electro-spark alloying (ESA) method is a quite widespread technology for the enhancement of wear resistance and lifespan of critical parts in the industry [1,2]. ESA is based on the chemical mixing of the melted substrate with the deposited material transferred from the electrode during spark gas discharge. The method has multiple advantages, such as high adhesion [3,4], the possibility of local treatment of the surface, low thermal impact on the substrate [5,6], absence of strict requirements for surface preparation prior to the deposition, high transportability, and simplicity of implementation [4]. The deposition process, in the easiest case, can be realized in the environment of the air [7].

Nevertheless, the ESA method has a number of disadvantages, such as a high content of structural defects (pores, microcracks) [8], high level of surface roughness [9], in some cases, contamination by oxygen (for example, if the process was conducted in an air environment), and the presence of substrate matter in the coating composition [10].

The ESA process can be optimized in multiple ways, including the application of protecting gas (argon, nitrogen) or liquid (alcohol, oil) environments, which allow one to improve the quality of the coatings [11,12,13]. Vacuum electro-spark alloying (VESA) is another promising modification of the process, but has some caveats of its own. On the one hand, coatings deposited in noble gas environments (He, Ar) and in vacuums have lower defect concentrations in comparison with ones deposited in air; on the other hand, pressure decrement leads to the decrease in the coating growth rate [14]. Research [15] shows that coatings made in vacuums are more chemically homogeneous and have higher corrosion resistance. In addition, the changes in the phase compositions of coatings were mentioned, resulting from the extended exposure to higher temperatures in the discharge region and a lower level of heat losses to the outer environment in a vacuum. Another direction of development of ESA technology is a combination of ESA with other surface strengthening and modification methods. We can mention researches on the additional treatment of electro-spark deposited layers using the micro-discharge oxidation method [16,17]. The results show that after the follow-up treatment, the microhardness of ESA coatings was increased by 80% and wear resistance was improved by 70%. Researches [18,19] are showing that laser treatment of ESA coatings contributes to the self-healing of pores and microcracks, increases microhardness by 20% and corrosion resistance by 30%, and decreases the coefficient of friction for more than two times. ESA treatment of titanium alloys using a steel electrode with subsequent surface carbonization leads to 100% higher hardness due to the increased content of titanium carbide in the surface layer [20]. Application of nitriding in plasma [21] as a post-treatment allows one to increase the material’s hardness by nearly 100%, as compared to the vanilla single-stage ESA technology. Another branch of ESA-related research is the fabrication of multilayered coatings using a combination of ESA and magnetron sputtering methods [22,23]. Magnetron sputtering allows one to improve the hardness by 1.5–3 times and corrosion resistance by 40% [23]. The application of the pulsed cathodic arc evaporation (PCAE) method is also promising in this regard [24,25,26]. Mechanical finishing of the ESA layer is another way to improve its tribological characteristics due to the reduction of surface roughness and change of the surface wear mechanism [23]. The combined post-treatment of ESA coatings by diamond polishing and ion-plasma sputtering of TiN coatings was discussed in [27].

The application of TiC-Ni electrodes is quite widespread in ESA technology [28,29,30]. TiC grains, which are forming during the treatment of steel by such electrodes in the near-surface layer, provide high hardness and wear resistance, and the solid solution of nickel in iron increases the strength and corrosion resistance of the substrate [31]. The addition of chromium increases the mechanical and tribological properties as well as the heat resistance of ESA coatings [30]. The introduction of ZrO_2_, Al_2_O_3_, NbC, WC, and other additives into TiC-Ni and TiC-NiCr electrodes allows one to increase the coating growth rate, hardness, and heat resistance as well as to decrease the coefficient of friction [2]. Earlier, we demonstrated that the introduction of Eu_2_O_3_ into the electrode composition leads to the increase in their strength [32], coating growth rate [33], mechanical/tribological properties, and corrosion resistance of the coatings [34,35]. The introduction of additional carbon, for example, from a reactive atmosphere, and the formation of diamond-like carbon allow one to reduce the friction coefficient and enhance the corrosion resistance of coatings [32]. Sputtering of the upper diamond-like carbon (DLC) layer on the arc-deposited base TiC coatings increased the hardness and elastic modulus by 60% and 20%, respectively [36].

The current study is aimed at research on coatings deposited by a combination of VESA and PCAE technologies using TiC-NiCr-Eu_2_O_3_ electrodes and steel 5140 as the substrate. The upper layer was formed by PCAE in the ethylene environment in order to increase the tribological performance of the whole coating due to the formation of the DLC-based layer.

## 2. Materials and Methods

TiC-NiCr-Eu_2_O_3_ electrodes of 4 × 4 × 50 and 10 × 10 × 75 mm size were manufactured for the VESA and PCAE processes using the powder metallurgy route. Mixing and mechanical activation of the powder mixture (81.0 wt.% TiC, 5.1 wt.% Ni, 8.2 wt.% Cr, 5.7 wt.% Eu_2_O_3_) were performed in a planetary ball mill “Aktivator-2S” with a jar radius of 0.040 m and carrier radius of 0.104 m. Milling was performed for 5 min at the velocity of 694 rotations per minute using steel milling bodies (balls) with a weight of 0.8 g and a radius of 0.003 m in the Ar environment. As-treated powder mixtures were compacted on a hydraulic press with a pressure of 200 MPa. Sintering was performed at 1450 °C for 60 min in the Al_2_O_3_ filler in a vacuum oven VE-3-16 (JSC "NPP VacETO", Moscow, Russia). Coatings were deposited on Ø30 × 5 mm polished discs of 5140 steel. Before the deposition, substrates were ultrasonicated in isopropanol for 5 min using the UZDN-2T device (LLC "NPP UKRROSPRIBOR", Sumy, Ukraine).

**Figure 1:**
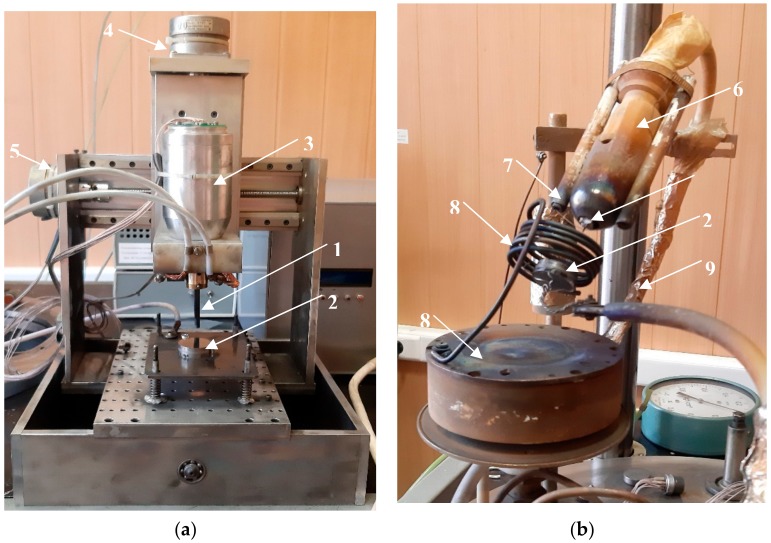
Appearance of blocks for vacuum electro-spark alloying (VESA) (**a**) and pulsed cathodic arc evaporation (PCAE) (**b**): 1—electrode; 2—substrate; 3—engine for electrode spinning; 4—engine for the moving of the whole block along the Y axis; 5—engine for the moving of the whole block along the X axis; 6—ceramic isolation; 7—holder of the ignition electrode; 8—anode; 9—supply and cooler leads.

Automated VESA processing and PCAE deposition were performed on a custom device based on the UVN-2M vacuum system (ZTO, Moscow, Russia) and two different blocks for deposition processing. Electro-spark coatings were fabricated using a three-coordinate VESA module (Figure 1a), designed for work in a vacuum.

VESA processing was carried out using the following parameters: Residual pressure of 10 Pa, normal polarity (electrode–anode), voltage of 100 V, pulse duration of 20 μs, pulse frequency of 100 Hz, 20 iterations, and rotational velocity of 1000 rounds/min.

For the PCAE process, we used a module with an originally-designed evaporator (Figure 1b). The ignition voltage and frequency were 15 kV and 10 Hz, and the arc voltage was oscillating between 160 and 200 V. Before the coating deposition, substrates were cleaned in a vacuum by application of a 2 kV bias voltage in Ar for 5 min. At first, a PCAE layer was deposited in Ar (99.9995%) on top of the VESA coating for 10 min, then the upper layer was deposited for 10 min with the injection of C_2_H_4_ (99.95%) in a chamber. The pressure of working gases was 0.4 Pa, and residual pressure was 4 × 10^−3^ Pa.

The morphological, elemental, and phase compositions of coatings were investigated by scanning electron microscopy (SEM) and energy-dispersive spectroscopy (EDS) using a Hitachi S-3400N microscope with a Noran 7 Thermo add-on. X-ray diffraction analysis (XRD) was performed on the AXS D8 AD-VANCE device (Bruker, Sulgen, Germany) using Cu–Ka radiation. Raman spectroscopy was performed using the NTEGRA NT-MDT (Moscow, Russia) installation equipped with a red laser (633 nm wavelength). Mechanical properties of coatings, such as hardness (H), elastic modulus (E), and elastic recovery (W), were measured on a Nano Hardness Tester (CSM Instruments, Portland, OR, USA) equipped with a Berkovich diamond indenter. Transversal cross-cuts of the VESA samples were tested at a 10 mN load, whereas the PCAE coatings were examined by indentation from the top with a 4 mN load. Tribological testing was performed using a pin-on-disc scheme on the Tribometer CSM Instruments device. The samples were tested in contact with an Al_2_O_3_ ball with a diameter of 6 mm at room temperature in air and in liquid (distilled water) with a linear velocity of 10 cm/s and perpendicular load of 5 N. In order to examine the wear tracks and determine the surface roughness, an optical profiler “WYKO NT 110” (“Veeco Instruments Inc.”, Plainview, NY, USA) was used. The wear of the counter-body and of the coatings was calculated by the formula mentioned in [37]. The electrochemical properties of the coatings were evaluated with the use of three-electrode cells with a VoltaLab 50 (Radiometer Analytical, Villerbanne Cedex, France) potentiostat. The tests were conducted in a 1 N H_2_SO_4_ solution using the Ag/AgCl reference electrode and auxiliary Pt electrode. All potentials were recounted in relation to the standard hydrogen electrode. The density of the corrosion current (i_cor_) was calculated using the Tafel formula.

## 3. Results 

### 3.1. Elemental Analysis of the Coatings

The elemental compositions of the substrates and of each of the layers in the multi-layered coatings are presented in Table 1.

A total of 21.5 wt. % of Fe (the substrate material) is present in the VESA coatings. The Ti to C ratio is close to the stoichiometric composition (1:1), whereas the Ni to Cr ratio is 1:2. The Eu concentration is 3.9 wt. %. The PCAE coating deposited in argon features reduced iron content (1.6 wt. %) due to the specifics of the deposition process. The contents of the main elements, Ti, C, Ni, and Cr, as compared to the VESA coating increased by up to 2.2, 1.4, 0.6, and 2.9 times, respectively.

### 3.2. Microstructure and Phase Composition of the Coatings

**Figure 2:**
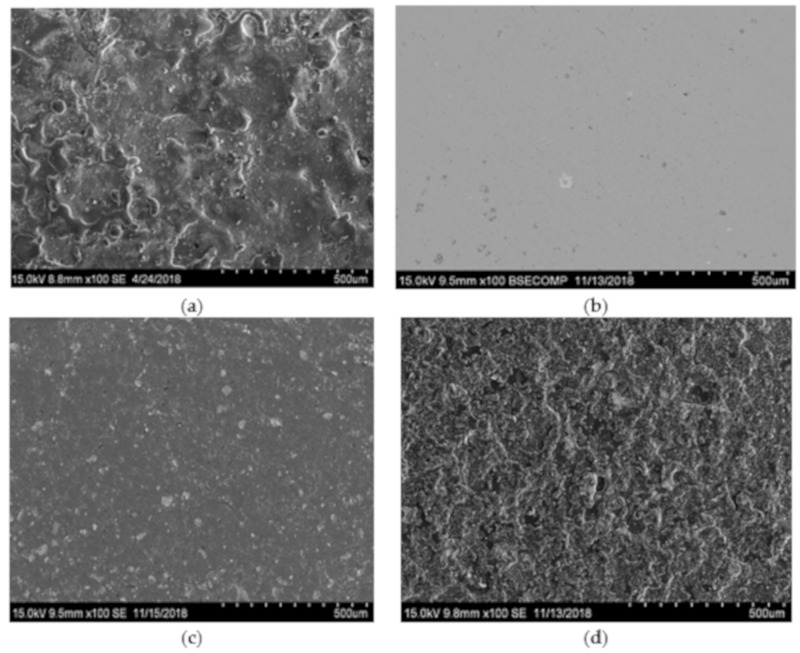
Scanning electron microscopy (SEM) top-view images of the coating surface: VESA (**a**), PCAE in Ar (**b**), PCAE in C_2_H_4_ (**c**), and VESA–PCAE (**d**).

Figure 2 provides typical SEM images of the surfaces of coatings deposited by VESA and PCAE in Ar and C_2_H_4_, and the coating fabricated by the VESA–PCAE technique.

**Figure 3:**
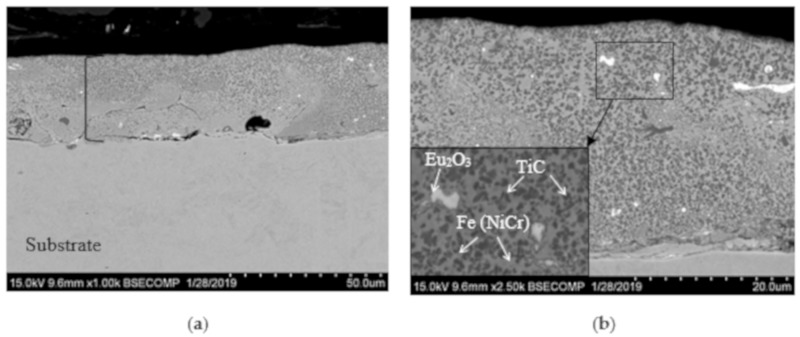

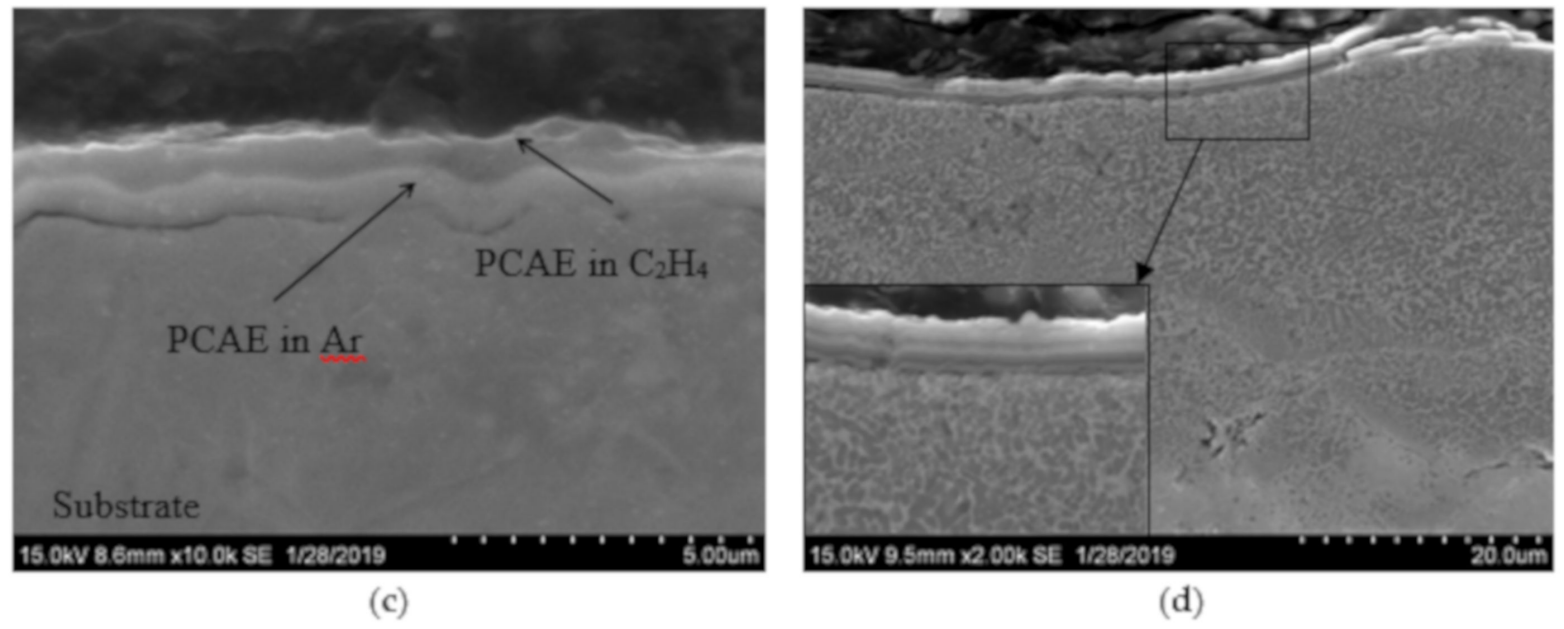
SEM cross-section images of the VESA (**a**,**b**), PCAE in Ar and C_2_H_4_ (**c**), and VESA–PCAE (**d**) coatings.

On the surface of the VESA coating, small quantities of microcracks, droplets, and condensed metallic spatter can be observed. The transverse fractures of single- and multilayered coatings (Figure 3) demonstrate that VESA coatings contain TiC grains, regions of solid solutions of Ni and Cr in Fe, and Eu_2_O_3_ grains, which are present both on the substrate–coating interface and in the coating’s volume.

In the case of the single-layer coatings, the sizes of TiC and Eu_2_O_3_ grains were in the 0.2–1.5 μm and 0.2–2.0 μm ranges, respectively. In the multilayer coatings, the sizes of the TiC grains were similar, but the Eu_2_O_3_ grains were finer (0.1–0.5 μm). The VESA–PCAE sample consisted of a lower VESA layer with a thickness of 28 μm, middle PCAE layer deposited in Ar (0.8 μm), and upper PCAE layer (deposited in C_2_H_4_) with a similar thickness of 0.8 μm.

**Figure 4:**
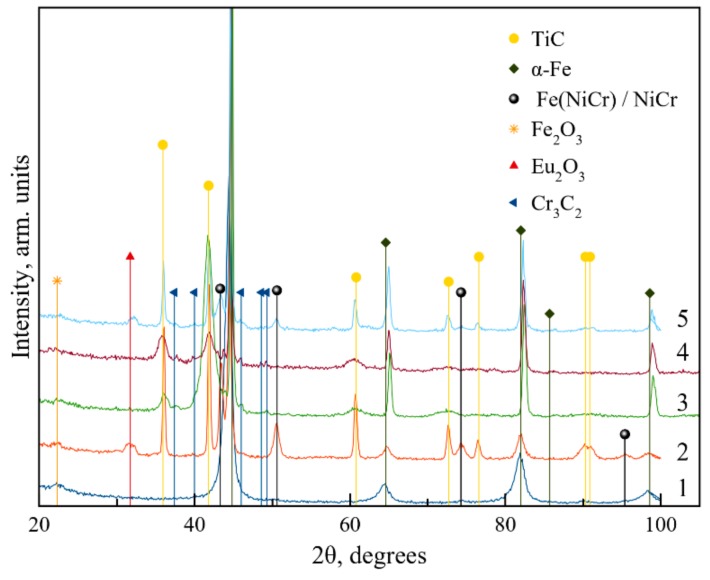
X-ray diffraction (XRD) patterns from the steel substrate (1) and the coatings deposited by VESA (2), by PCAE in Ar (3) and in C_2_H_4_ (4), and by VESA–PCAE (5).

Figure 4 provides the XRD patterns for the steel 5140 substrate, single-layer VESA coatings, PCAE coatings (deposited in Ar and C_2_H_4_), and multilayer VESA–PCAE coatings.

All coatings featured peaks corresponding to (110), (200), (220), (311), (222), and (400) lines of the TiC-based face centered cubic phase. The PCAE coatings deposited in Ar and C_2_H_4_ featured TiC peaks (Card No. 89-3828 of International Center for Diffraction Data (ICCD)) corresponding to planes (111), (200), (220), and (311). In the samples obtained by VESA and VESA–PCAE, the peaks of a solid solution of Ni and Cr in Fe (Fe (NiCr)) (ICCD 52-0513) are presented on the XRD patterns. Samples 3 and 4 demonstrate that upon the deposition, Cr_3_C_2_ (ICCD 65-0897) grains are formed, corresponding to planes (211), (301), (121), and (221). The peak at 2Θ = 32.1 is specific to Eu_2_O_3_ (ICCD 65-3182).

The crystallite size was determined using the Scherrer formula. The sizes of the TiC crystals calculated from the broadening of lines (111), (200), and (220) for the VESA sample were 22, 23, and 16 nm, respectively. For the PCAE (Ar) coating, the sizes of the crystals are lower: 9, 11, and 7 nm, respectively. For the PCAE (C_2_H_4_) coating, the observed TiC sizes further decrease to 4–7 nm, accompanied by the formation of Cr_3_C_2_ crystals with sizes of 25–40 nm. The peaks for VESA–PCAE contain all of the peaks observed for single-layer coatings.

**Figure 5:**
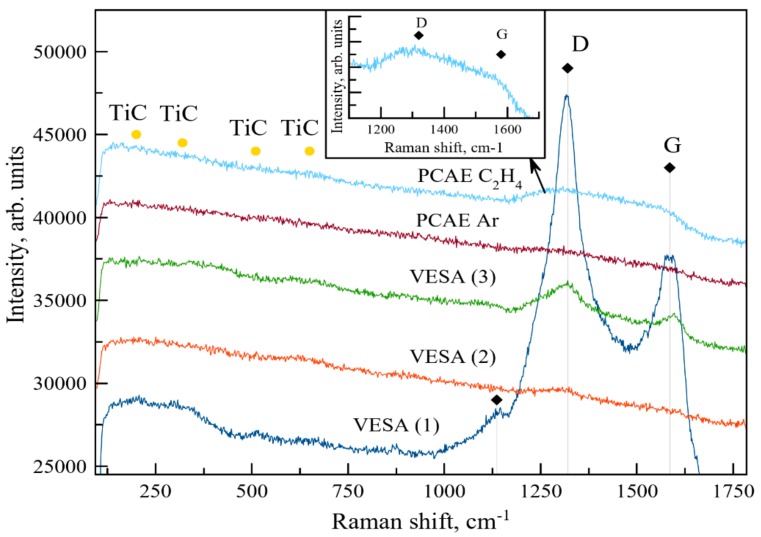
Raman spectra of the coatings. The insert provides the Raman spectra of the PCAE C_2_H_4_ coating at ×2 magnification.

Raman spectra for the VESA and PCAE coatings are shown in Figure 5.

The Raman spectra for all coatings contained peaks corresponding to TiC (200, 320, 510, and 650 cm^−1^ [38,39]). The exact shapes of the spectra for the VESA coating depended on the place of analysis due to the heterogeneity of the spark-deposited layer. Therefore, Figure 5 provides spectra for three specific regions of the VESA coating. In addition to TiC, the VESA samples also demonstrated carbon peaks at positions 1140, 1320, and 1585 cm^−1^ [40,41]. The shapes of D and G mean that crystalline graphite forms on the surface of the VESA sample [41]. In the case of PCAE coatings deposited in C_2_H_4_, the shapes and locations of peaks suggest the formation of a diamond-like carbon (DLC) phase [41].

### 3.3. Mechanical Properties of the Coatings

The mechanical characteristics of the coatings are shown in Table 2.

The VESA coating was characterized by a hardness of H = 12.2 GPa, Young’s modulus of E = 273 GPa, and elastic recovery of W = 37%. The VESA coatings provided a threefold increase in the hardness and elastic recovery of the substrate. The intermediate layer of PCAE (Ar) had the highest mechanical properties: H = 30.4 GPa, E = 286 GPa, and W = 79%. Reactive deposition (in C_2_H_4_) lead to a decrease in H and E values by 4.5 and 4 times, respectively, along with a decrease of W to 68%.

### 3.4. Tribological Characterization of the Coatings

**Figure 6:**
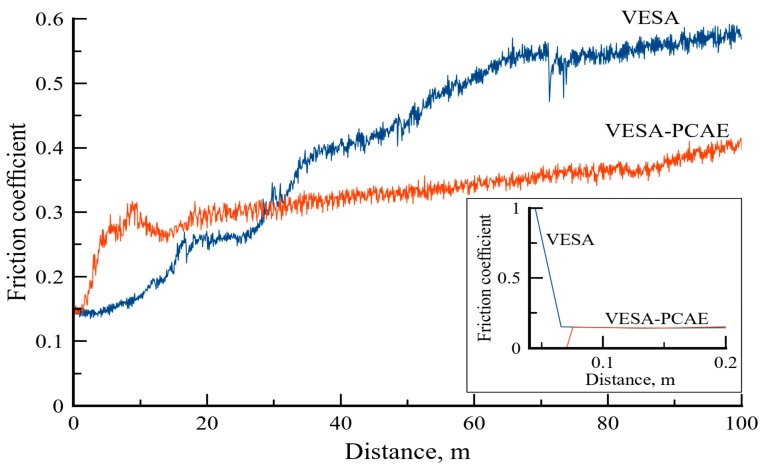
The friction coefficient of coatings in air. The insert demonstrates the initial coefficient of friction.

According to the results of tribological testing at room temperature, a coefficient of friction (*f*) for PCAE (Ar) smoothly grew in the 0–70 m range with an average *f* value of 0.64. After 70 m, *f* grew to 0.8, which is the coefficient of friction of the substrate. The PCAE (C_2_H_4_) demonstrated a stable *f* = 0.36 in the range of 0–46 m. Afterward, the value jumps up due to the wearing of the coating and contact between the counter-body and the substrate [37]. The friction data for the single-layer and multilayer coatings are provided in Figure 6.

**Figure 7:**
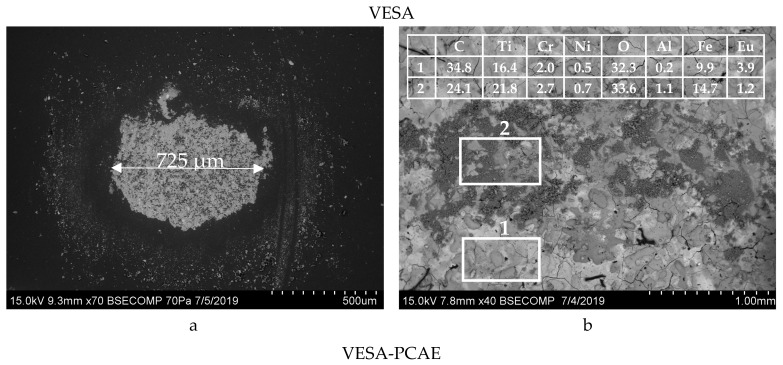

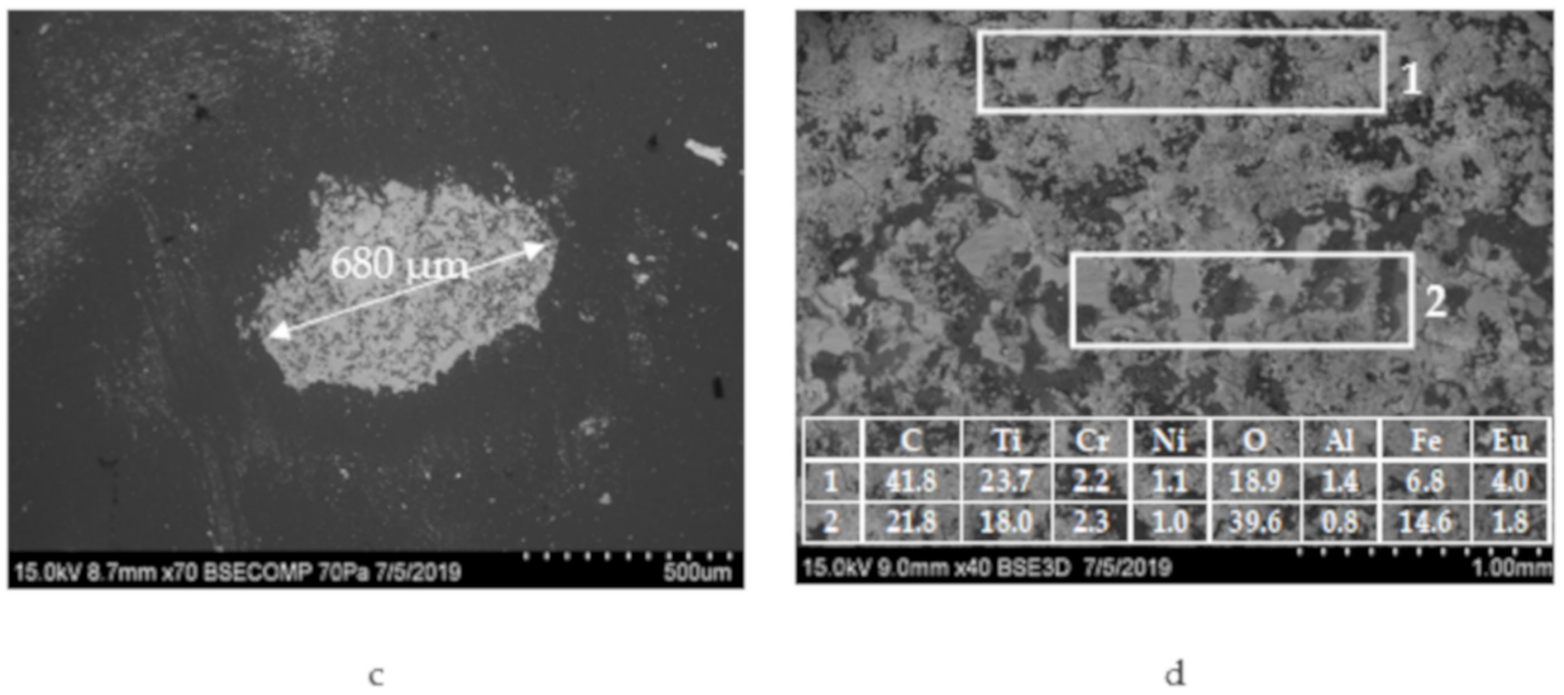
SEM images of the wear on counter-body materials (**a**,**c**) and wear tracks on coatings (**b**,**d**) in air. The insets show the energy-dispersive spectroscopy (EDS) elemental analysis of wear tracks (at. %).

It can be seen that *f* of the VESA coating grew gradually (from 0.14 to 0.58) during the whole distance of 100 m. The average coefficient of friction value was 0.41. The multilayer coating had a stable coefficient of friction equal to 0.33. Interestingly, the VESA–PCAE coatings demonstrated a higher initial friction coefficient as compared to the VESA samples (insert in Figure 6). The SEM investigation (Figure 7) of the wear tracks on the coatings and counter-bodies suggests the higher wear resistance of VESA–PCAE coating.

In the case of the single-layer coatings, the wear of the Al_2_O_3_ ball was 4.53 × 10^–5^ mm^3^/N/m. Wear products (oxides of iron and titanium) can be observed in the tribocontact zone, but the coating was not fully worn out. Despite its lower coefficient of friction, the PCAE layer of a multi-layered coating was fully worn out by the end of the test. DLC-containing wear products from the upper layer were present in the tribocontact zone and decreased the coefficient of friction and counter-body wear to 3.5 × 10^–5^ mm^3^/N/m.

**Figure 8:**
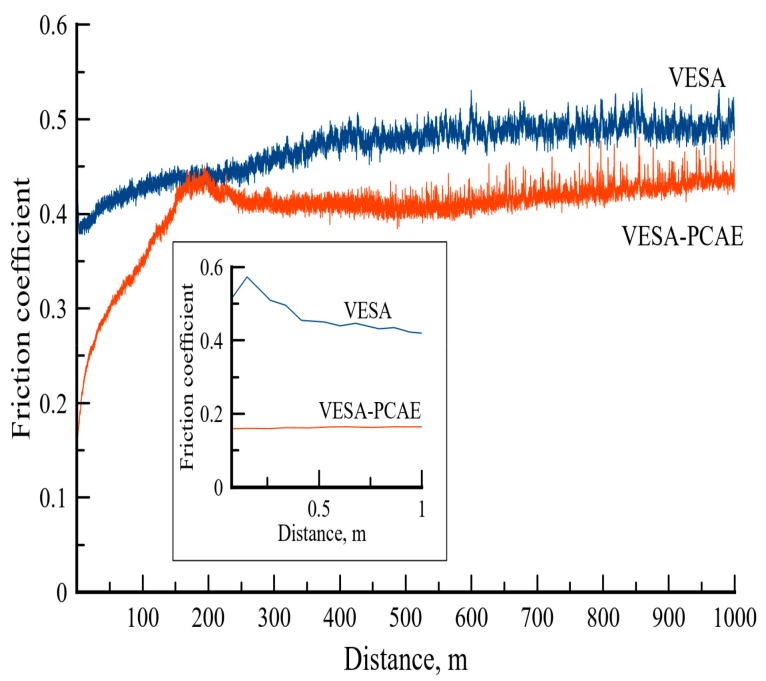
The friction coefficients of coatings in distilled water. The insert demonstrates the initial friction coefficients.

The tribological characteristics were also examined in distilled water (Figure 8).

**Figure 9:**
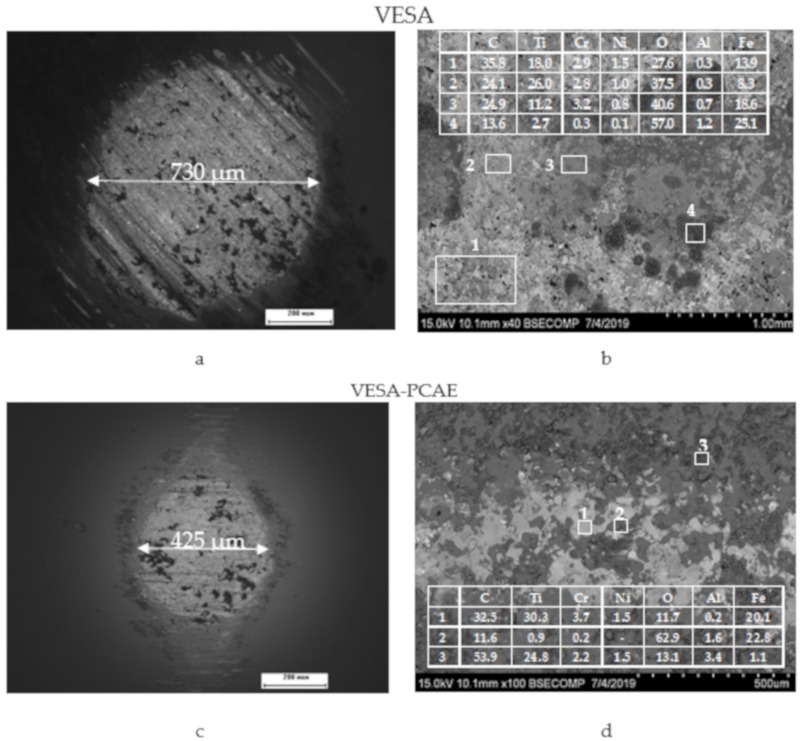
Results of the investigation of the wear of the counter-bodies (**a**,**c**) and wear tracks (**b**,**d**) in distilled water. The insets show the EDS elemental analysis of wear tracks (at. %).

The VESA coating had a relatively high initial *f* = 0.55, which then decreased to 0.38. In the 400–1000 m distance, the friction coefficient stabilized at a value of 0.5. The VESA–PCAE coating had a low initial *f* = 0.18. At the distance of 200 m, the friction coefficient experienced a sharp increase, but then it decreased again and remained stable in the distance of 250–1000 m. The average *f* value for the VESA–PCAE coating was 0.40, which is 15% lower than the friction coefficient of the single-layer coating (0.47). The wear zones of the counter-bodies after the contact with VESA and VESA–PCAE coatings are shown in Figure 9.

In the case of the VESA sample, the specific wear of the alumina ball was 4.67 × 10^−6^ mm^3^/N/m. In contact with the multilayer coating, the wear of the counter-body was much lower (0.54 × 10^−6^ mm^3^/N/m). The VESA coating experienced little to no wear, although some wear products (Fe_x_O_x_) were observed in the tribocontact zone. Similarly to the results of friction in the air, the upper layer of the VESA–PCAE coating was worn down, and the wear products decreased the friction coefficient. The examination of the tribocontact zone revealed the presence of carbon, Fe, and Al oxides along with small shards of VESA and PCAE coatings.

### 3.5. Electrochemical Properties of the Coatings

**Figure 10:**
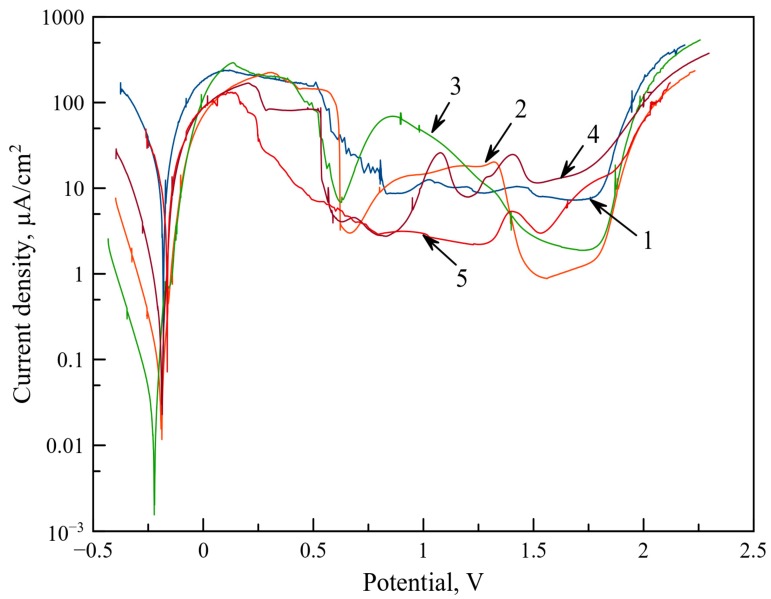
Polarization curves for the coatings in semi-logarithmic coordinates: 1—VESA; 2—PCAE Ar; 3—PCAE C_2_H_4_; 4—VESA–PCAE; 5—steel substrate.

The data pertaining to the electrochemical properties of single-layer and multi-layer coatings are provided in Figure 10 as polarization curves in semi-logarithmic coordinates.

All coatings were passivated in sulphuric acid. Under anode polarization in the 0.5–1.5 V diapason, all coatings demonstrated an activational current density peak, related to the oxidation of nickel, TiC, and Cr_3_C_2_, with the subsequent isolation of the surface from the electrolyte by the as-formed oxide layers. At potentials above 1.75 V, all coatings experienced the electrical breakdown of the passivating film. The free corrosion potential φ and density of corrosion current i_cor_ are provided in Table 3.

The stable free corrosion potentials were –170, –200, –230, and –190 mV for the coatings of VESA, PCAE deposited in Ar and C_2_H_4_, and VESA–PCAE, respectively. The density of corrosion current for the VESA coating was 3500 μA/cm^2^, which is close to the iron corrosion current density (4500 μA/cm^2^). The i_cor_ value for the PCAE (Ar) coating was 54 μA/cm^2^_,_ and for the PCAE (C_2_H_4_) coating, it was 12 μA/cm^2^. Thus, the PCAE deposition of the upper DLC layer reduced the current density of the corrosion by 4.5 times. The multilayered VESA–PCAE coatings demonstrated the current density at the level of 150 μA/cm^2^, which is 23 times smaller than the mono-layer VESA samples.

## 4. Discussion

The VESA coating contains up to 22 wt. % of Fe (the substrate material). The Ti to C ratio is close to the stoichiometric composition (1:1), whereas the Ni to Cr ratio is 1:2. Eu concentration is 3.9 wt. %. The PCAE coating deposited in argon features a reduced iron content (1.6 wt. %) due to the specifics of the deposition process. The contents of the main elements Ti, C, Ni, and Cr, as compared to the VESA coating, increased by up to 2.2, 1.4, 0.6, and 2.9 times, respectively. The PCAE coating produced in C_2_H_4_ featured the highest carbon content of 87.2 wt. %, probably due to the additional influx of C atoms from reactive C_2_H_4_. The surplus of carbon atoms should lead to the formation of amorphous diamond-like carbon in addition to TiC. This coating is also characterized by the reduced concentration of metallic elements.

The VESA coating had a characteristic microstructure with the presence of microcracks, droplets, and condensed metallic spatter [8]. All of these defects are deleterious for mechanical properties, tribological performance, and corrosion resistance. The emergence of cracks can be caused by thermal stresses in the coating, which is basically unavoidable in VESA coatings. In contrast, the PCAE Ar sample showed a uniform structure. The droplet phase, which is specific for that deposition method, was practically absent both in the volume and on the surface of the coating. However, the coating features relatively prolific delamination (up to 2%). The fundamental benefit of the VESA–PCAE technique lies in the fact that the various layers in the coatings can compensate for each other’s drawbacks. For example, in the layered coatings produced by VESA–PCAE, the upper layer inherited the topography of the lower layer (with its specific microcracks and droplets of molten metal). However, the upper layer itself was extremely uniform and contained no defects or delamination areas. This is probably caused by the favorable surface morphology of the relatively rough basic VESA layer, which promotes the adhesion between the layers within a coating.

It is known that VESA coatings based on TiC-Ni have relatively low hardness in the range of 13.9–15 GPa [30,42]. PCAE coatings have higher mechanical properties. For example, the hardness of single-layer arc-deposited TiC-NiCr-Eu_2_O_3_ coatings is in the range of 17–21 GPa [34]. However, the deposition of the upper layer in ethylene produces a trade-off between the mechanical, tribological, and electrochemical properties of the multilayered coating. In general, the increased concentration of carbon results in the decreased hardness of the coating, presumably due to the reduction of sp^3^ bonds and the prevalence of sp^2^ bonds [43]. This might be related to the alloying of the diamond-like carbon phase by Ti and Cr atoms, which decreases the ratio of sp^3^ bonds and the hardness of the phase. We cannot rule out the influence of the formation of new phases, such as Cr_3_C_2_ (which was confirmed by XRD).

The results of tribological tests suggest that the deposition of VESA–PCAE coatings results in the reduction of the friction coefficient during the wear of the base layer, both in air and in distilled water. In multilayer coatings, the upper PCAE layer provides a low friction coefficient and the lower VESA layer has a high wear resistance.

The corrosion potentials of all single-layer coatings deposited on the steel substrates are equal to the free corrosion potential of iron (190 V). This is related to the spread of the electrolyte through the cracks and defects of the coating towards the substrate with the subsequent active dissolution of the iron. The lowest corrosion current density (12 μA/cm^2^) was achieved for the coating sputtered in ethylene, which might be related to the positive influence of chromium carbide and increased carbon content [44]. The I_cor_ of the multi-layered coating decreased by at least 20 times in comparison with the basic VESA coating. This indicates an improvement in the corrosion resistance of VESA coatings due to the deposition of the upper layer with high carbon content. The decrease of the corrosion current density might also result from the healing of cracks and other defects of the spark-deposited layer, as was demonstrated in [45].

Therefore, the design of the multilayered VESA–PCAE coatings allows for the custom-fitting of the coatings to the intended application condition. If the main risk factors are wear or electrochemical oxidation, the reactive deposition of the DLC-based upper layer is advisable, whereas in order to obtain the highest possible mechanical properties, one must opt for the deposition of the upper layer in an inert atmosphere.

## 5. Conclusions

TiCNiCr-Eu_2_O_3_ coatings were produced by VESA and PCAE in argon and ethylene, as well as by the combined VESA–PCAE technology. The coatings were comprised of TiC and Fe (NiCr) in the case of VESA and VESA–PCAE deposition and of NiCr in the case of PCAE, Cr_3_C_2_ (for PCAE in C_2_H_4_ and VESA–PCAE), and Eu_2_O_3_. The VESA coating was characterized by relatively low hardness (H = 12.2 GPa); however, this property could be fortified by the deposition of an intermediate PCAE layer in Ar (H = 30 GPa). The deposition of the DLC-containing upper layer of PCAE reduced the friction coefficient by at least 50% in air and 20% in distilled water. The upper PCAE layer, deposited in C_2_H_4_, provided a lower corrosion current density in the 1 N solution of H_2_SO_4_ (150 versus 3500 μA/cm^2^).

For the first time, the two-stage VESA–PCAE technology was applied for the deposition of multilayered coatings based on TiC with an NiCr binder. Such coatings surpass the single-layered VESA samples by a large margin when it comes to tribological characteristics and corrosion resistance. Although the strength of the upper layer deposited in C_2_H_4_ was somewhat lower, some multilayered coatings demonstrated a lower coefficient of friction in air and distilled water due to the formation of the DLC phase in the upper layer. Due to the tight sealing of the defects in the base layer by the upper PCAE layer, the corrosion current decreases. Therefore, the VESA–PCAE technology can be applied for the increase of wear and corrosion resistance for parts which are used in the air or in corrosive environments.

The potential areas of application of multi-layered TiC-NiCr-Eu_2_O_3_-devised coatings deposited via the VESA–PCAE technique are the automotive and aerospace industries. The electrodes and VESA–PCAE technique which were approbated in this work can be applied for the increase of the performance and lifespan of parts which are usually treated by the single-stage ESA technique, such as crankshafts, camshafts, valves and pistons of internal combustion engines, edges and roots of turbine blades, parts of pumps, etc.

## Figures and Tables

**Table 1:** Chemical composition of VESA and PCAE coatings.

Material	Concentration wt. %										
	Ti	C	Ni	Al	Fe	Si	Mn	Cr	Eu	O	N
VESA	21.7	26.3	1.1	0	21.5	0	0	2.2	3.9	21.2	2.1
PCAE Ar	47.3	37.3	1.8	0.2	1.6	0	0	6.5	1.1	0	–
PCAE C_2_H_4_	9.4	87.2	0.8	0.9	0.3	0	0	1.3	0	0	0
Substrate	–	9.0	–	0.1	89.0	0.6	0.6	0.7	–	–	–

**Table 2:** The mechanical properties of the coatings and substrate.

Material	H, GPa	E, GPa	W, %
VESA (cross section)	12.2	273	37
PCAE Ar	30.4	286	79
PCAE C_2_H_4_	6.8	71	68
Substrate	3.7	223	12

**Table 3:** The electrochemical properties of the investigated coatings.

Material	E, mV	i_cor_, μA/cm^2^
VESA	−170	3500
PCAE Ar	−200	54
PCAE C_2_H_4_	−230	12
VESA-PCAE	−190	150
Substrate	−190	4500
